# Swept-Source Optical Coherence Tomography Thresholds in Differentiating Clinical Outcomes in a Real-World Cohort of Treatment-Naïve Multiple Sclerosis Patients

**DOI:** 10.3390/brainsci13040591

**Published:** 2023-03-31

**Authors:** Łukasz Rzepiński, Jan Kucharczuk, Magda Tkaczyńska, Vincenzo Parisi, Andrzej Grzybowski

**Affiliations:** 1 Department of Neurology, 10th Military Research Hospital and Polyclinic, Powstańców Warszawy 5, 85-681 Bydgoszcz, Poland; 2 Sanitas—Neurology Outpatient Clinic, Dworcowa 110, 85-010 Bydgoszcz, Poland; 3 Department of Ophthalmology, 10th Military Research Hospital and Polyclinic, Powstańców Warszawy 5, 85-681 Bydgoszcz, Poland; 4 Department of Surgery, 10th Military Research Hospital and Polyclinic, Powstańców Warszawy 5, 85-681 Bydgoszcz, Poland; 5 IRCCS—Fondazione Bietti, Via Livenza 3, 00198 Rome, Italy; 6 Department of Ophthalmology, University of Warmia and Mazury, Żołnierska 18, 10-561 Olsztyn, Poland; 7 Institute for Research in Ophthalmology, Foundation for Ophthalmology Development, Mickiewicza 24/3B, 60-836 Poznan, Poland

**Keywords:** multiple sclerosis, optical coherence tomography, GCIPL, pRNFL, disability progression

## Abstract

This study aimed to determine whether peripapillary retinal nerve fiber layer (pRNFL) and ganglion cell–inner plexiform layer (GCIPL) thickness thresholds for single-time-point swept-source optical coherence tomography (SS-OCT) measures can differentiate the clinical outcomes of treatment-naïve people with multiple sclerosis (pwMS). A total of 275 patients with the clinically isolated syndrome (n = 23), benign MS (n = 8), relapsing–remitting MS (n = 185), secondary progressive MS (n = 28), primary progressive MS (n = 31), and with no history of optic neuritis were included. The mean Expanded Disability Status Scale (EDSS) score was 3.0 ± 1.6. The cut-off values of pRNFL (87 µm and 88 µm) and GCIPL (70 µm) thicknesses have been adopted from previous studies using spectral-domain OCT. PwMS with pRNFL ≤87 µm and ≤88 µm had a longer disease duration, more advanced disability, and more frequently progressive MS variants compared to those with greater pRNFL thicknesses. In distinguishing pwMS with disability greater than or equal to the mean EDSS score (EDSS ≥ 3) from those with less severe disability, GCIPL thickness <70 µm had the highest sensitivity, while pRNFL thickness ≤87 µm had the greatest specificity. The optimal cut-off values differentiating patients with EDSS ≥ 3 from those with less severe disability was 63 µm for GCIPL thickness and 93.5 µm for pRNFL thickness. In conclusion, pRNFL and GCIPL thickness thresholds for single-time-point SS-OCT measurements may be helpful in differentiating the disability status of treatment-naïve pwMS.

## 1. Introduction

Multiple sclerosis (MS) is an acquired, immune-mediated disease resulting in the formation of demyelinating lesions and subsequent neuroaxonal loss within the central nervous system (CNS) [1]. Approximately 85–90% of patients experience the first attack in the form of clinically isolated syndrome (CIS), being subsequently diagnosed with relapsing–remitting MS (RRMS) [2]. In most cases, RRMS characterized by pathogenic immune responses is followed by a phase of insidious disability accrual with underlying neurodegenerative processes, termed as secondary progressive MS (SPMS) [1,3]. Predominant neuro-axonal loss in about 10–15% of patients leads to a gradual disability progression from the disease onset, referred to as primary progressive MS (PPMS) [2,4,5]. A relatively mild disease course, called benign MS (BNMS), is observed in a subset of patients [6].

The inflammatory and neurodegenerative mechanisms are the fundamental drivers of clinical progression in MS, and both of them may be visualized by using paraclinical tools. Optical coherence tomography (OCT) is a non-invasive, rapid, and reproducible high-resolution imaging technology to assess distinct retinal layers [7]. As the retina shows similarities in its functionality, anatomical structure, and response to damage to the brain and spinal cord, it can be viewed as a window into the CNS [8,9]. The use of OCT enables the quantification of axonal loss by measuring peripapillary retinal nerve fiber layer (pRNFL) thickness and neuronal damage by measuring ganglion cell–inner plexiform layer (GCIPL) thickness and has, therefore, been widely applied in the evaluation of people with MS (pwMS) [10].

However, OCT has not been included in the diagnostic criteria for MS and the criteria for no evidence of disease activity [2,11]. Significantly, the paraclinical tools incorporated in MS diagnostic criteria precisely define the cut-off values, e.g., one or more demyelinating lesions in magnetic resonance imaging (MRI) in at least two of four CNS areas (to demonstrate dissemination in space) or more than two oligoclonal bands in the cerebrospinal fluid (for dissemination in time) [2]. Thus, identifying thresholds for OCT measures seems to be particularly valuable for strengthening its role in the diagnostic approach and monitoring of pwMS.

To date, few studies have defined specific cut-off values for pRNFL and GCIPL thickness for predicting MS-related disability progression within subsequent years. For example, Martinez-Lapiscina et al. found that pwMS with baseline pRNFL thickness ≤87 μm or ≤88 μm (measured by Cirrus and Spectralis OCT devices, respectively) in non-optic neuritis (non-ON) eyes had double the risk of long-term disability accrual [12]. Lambe et al. showed that single-time-point GCIPL thickness <70 μm in non-ON eyes was associated with a four-fold increase in odds of significant meaningful disability worsening in pwMS over a 10-year follow-up period [13]. Finally, Schurz and colleagues revealed that baseline thicknesses of GCIPL < 77 µm and pRNFL ≤ 88 µm in RRMS patients were associated with an increased risk of long-term disability progression [14].

Although these studies used a follow-up design, they were carried out in selected cohorts of patients—mostly including those with RRMS undergoing disease-modifying treatments (DMTs), using different methodologies, various spectral-domain OCT (SD-OCT) devices and not employing the newer generation of swept-source OCT (SS-OCT) systems [12,13,14]. The introduction of SS-OCT has improved the wavelength and scanning speed, thus enabling faster acquisition times, greater sensitivity, and lower imaging artifacts compared to SD-OCT [15]. In everyday practice, however, clinicians rarely have the opportunity to monitor subsequent SS-OCT measures and relate the findings to long-term disability progression of pwMS.

Our previous study demonstrated significant variant-dependent differences in SD-OCT measurements and a pronounced impact of optic neuritis (ON) history on inner retinal layers thickness loss in treatment-naïve pwMS [16]. Importantly, it has been shown that the use of DMTs, especially those of high effectivity, in addition to generating immune reconstitution, can also reduce retinal layers thinning [17,18,19]. Therefore, by eliminating the influence of DMTs and ON history, retinal thinning found in OCT becomes more dependent on MS-related neurodegeneration.

So far, the usefulness of retinal layer thickness thresholds in SS-OCT has not been assessed in a cohort of treatment-naïve patients with different disease types. Moreover, little is known about the utility of single-time-point SS-OCT measurements in distinguishing MS clinical outcomes. Therefore, we aimed to evaluate whether pRNFL and GCIPL thickness thresholds for single-time-point SS-OCT measurements can differentiate clinical outcomes in a real-world cohort of treatment-naïve pwMS without ON history.

Raising such objectives can contribute to expanding knowledge in the following areas:

- promoting the usefulness of SS-OCT in optimizing the evaluation of pwMS,
- indicating the potential significance of single-time-point SS-OCT measurements in non-ON eyes of treatment-naïve pwMS,
- searching for optimal pRNFL and GCIPL thickness thresholds to distinguish MS types,
- searching for optimal pRNFL and GCIPL thickness thresholds for discriminating non-ON eyes of CIS patients from non-ON eyes of pwMS, and
- the use of pRNFL and GCIPL thickness thresholds in differentiating the disability status of pwMS.

## 2. Materials and Methods

### 2.1. Study Design and Participants

We conducted a cross-sectional study. The study population consisted of 408 pwMS (273 females, 25 CIS patients) with a mean age of 39.5 ± 11.9 years, recruited consecutively and examined at an ophthalmological outpatient clinic. Inclusion criteria were diagnosis of CIS or MS according to the revised 2017 McDonald criteria and aged 18 years or older [2]. The exclusion criteria were a history of ON in any eye, the presence of CNS disorders other than MS, the use of DMTs, uncontrolled hypertension, diabetes, media opacity, glaucoma, retinal or choroidal diseases, severe nystagmus, or impaired eye movement that prohibits proper eye fixation, and any eye or optic nerve pathology that could affect SS-OCT measures.

The following clinical and demographic variables were collected from available medical records: age, sex, MS variant, disease duration, ON history, and use of DMTs. MS types at the time of the evaluation were classified as RRMS, SPMS, PPMS (according to the Lublin and Reingold classification), or BNMS [20]. BNMS was defined as an Expanded Disability Status Scale (EDSS) score ≤3 measured after a disease duration of at least 15 years [21].

As the history of ON has the most pronounced impact on the subsequent retinal atrophy, only non-ON eyes were qualified for further analysis [16,22]. To identify subclinical ON, we used a diagnostic approach based on interocular asymmetry with cut-off values of ≥9 µm for pRNFL and ≥6 µm for GCIPL thickness as previously described by Xu et al. [23]. Thicknesses of pRNFL and GCIPL were presented for the right and left eye to exclude interocular differences suggestive of ON, and then calculated as the mean value for both eyes for further statistical analysis. As a history of clinical or subclinical ON in both eyes may not reveal meaningful interocular differences in OCT measures, all patients with a history or parametric OCT indicators of ON were excluded from further evaluation. A similar selection method was used in patients whose distinct retinal layers in one eye could not be reliably assessed due to ophthalmic abnormalities.

Such an assumption allowed us to avoid the alleged decrease in the thicknesses of pRNFL and GCIPL, which were not related to MS-specific neurodegenerative processes. Furthermore, it has been suggested that ON in one eye might have a confounding effect on the retinal layer thickness of the non-ON eye through retrograde axonal degeneration due to the connection of both optic nerves via the optic chiasm [18,24]. These data are particularly important when analyzing the impact of single-time-point SS-OCT measurement and may be ignored in long-term monitoring of inner retinal layers atrophy.

All eligible participants underwent disability assessment on the EDSS on the day of the SS-OCT examination [25]. To determine the diagnostic accuracy of the adopted pRNFL and GCIPL thickness cut-off values in relation to disability status, the study group was divided into patients with a disability less than, greater than, or equal to the mean EDSS score.

Overall, 133 patients were excluded for the following reasons: 129 patients used DMTs, 108 patients had a history of ON (19 bilateral, non-simultaneous), two patients had glaucoma, one patient had severe nystagmus and one patient had media opacity affecting OCT measurements (the sum exceeds the total number of participants excluded due to overlap).

### 2.2. OCT Assessment

SS-OCT imaging (DRI OCT Triton, Topcon, Tokyo, Japan; software IMAGEnet6 for Triton Version1.02, version 1.34.19388) was performed by an experienced ophthalmologist after pupillary dilatation on both eyes of each patient at 100,000 A-scans per second. Scanning of the optic disc (512 A-scans, 258 B-scans) and macular region (512 A-scans, 128 B-scans) was performed in the automatically determined 6 mm × 6 mm squares using SMARTTrack function for stabilization. Measures of pRNFL thickness were obtained using the optic disc protocol covering the 3.4 mm ring scan automatically centered on the optic disc. The GCIPL thickness was measured as the combined ganglion cell layer and inner plexiform layer thickness obtained in the 3D Macula mode protocol automatically centered on the fovea. During image processing, automated segmentation of the pRNFL and GCIPL was performed using the manufacturer’s software with subsequent manual correction of obvious errors. For each patient, the repeatability of the OCT measures was checked for values <2 μm and the highest value was considered for statistical analysis [26]. Scans that did not fulfill the OSCAR-IB criteria were excluded from the further analyses [27,28]. OCT metrics were reported in accordance with the Advised Protocol for Optical Coherence Tomography Study Terminology and Elements (APOSTEL) recommendations [29].

### 2.3. Statistical Analysis

Regarding the epidemiological context of the research conducted, a patient (two eyes) was the subject (test entity) in the statistical analysis carried out. Demographic and clinical characteristics of the study cohort were compared using cut-off values for pRNFL thickness (87 μm and 88 μm, respectively) and GCIPL thickness (70 μm and 77 μm, respectively) previously determined by SD-OCT devices [12,13,14]. Therefore, those traits became dichotomous. Categorical traits were described through integers and percentages. Numerical variables were depicted by their mean, standard deviation (SD), 95% confidence interval (CI) with 3.92 standard error wide (2 × 1.96) and minimum-to-maximum values. For contingency tables a chi-squared test of independence or Fisher’s exact test (for small numbers) was performed. The normality of distribution was tested using the Shapiro–Wilk W test. The homogeneity of variances was assessed by Levene’s test. A multifactor analysis of variance (for normally distributed variables) or generalized linear models (for non-normally distributed ones) was conducted to assess differences in numerical variables between the study groups. For categorical independent variables, post hoc multiple comparisons were subsequently used in order to assume the least significant differences (LSD) in specific pairs of numerical results. A binary logistic regression model was fitted in order to estimate clinical or demographic predictors for the occurrence of the investigated OCT parameters beyond their relevant cut-off points. All the multifactor analysis of variance (ANOVA) and regression models were controlled for the study participants’ age, gender, disease duration, EDSS score and MS type. In order to assess their epidemiological characteristics and clinical usefulness in particular, receiver operating characteristic (ROC) curves were used, one for each cut-off point, e.g., pRNFL thickness ≤87 µm versus disability greater than or equal to the mean EDSS score. A proper series of epidemiological indicators was computed (area under the ROC curve, sensitivity, specificity, positive predictive value, negative predictive value, accuracy, and diagnostic odds ratio). The ROC curves were compared by using DeLong’s method [30,31]. A level of *p* < 0.05 was deemed statistically significant. All the statistical procedures were performed by using PQStat Software, version 1.8.4 (Poznan, Poland).

## 3. Results

A total of 275 treatment-naïve CIS and MS patients (550 eyes) without ON history was included. The clinical, demographic, and baseline OCT data of the patients are presented in Table 1. There were no meaningful differences in the mean thickness of pRNFL and GCIPL between the right and left eyes (Table 1).

### 3.1. GCIPL Thickness Cut-Off Points

When adopting a GCIPL thickness cut-off of 77 µm, the lower value was reached by 270 patients (98.2%), while the value ≥77 µm was found only in 5 patients (1.8%). On the other hand, for GCIPL thickness cut-off of 70 µm, values below versus equal or above were reached by 233 (84.7%) and 42 (15.3%) patients, respectively. Therefore, the cut-off value of 70 µm was adopted for further analysis. There were no significant differences between the analyzed subgroups in terms of patients’ sex, age, disease duration, EDSS score, and MS variant (*p* > 0.05) (Table 2 and Table 3).

### 3.2. pRNFL Thickness Cut-Off Points

In the study group, pRNFL thickness ≤87 µm was found in 74 patients (27%), while pRNFL thickness ≤88 was found in 79 patients (29%) (Table 2). Both values were adopted for further analysis. Statistically significant differences between MS variants were found for both pRNFL thickness cut-off points. None of the CIS patients had values ≤87 µm (Table 2). Patients with pRNFL thickness in non-ON eyes ≤87 µm and ≤88 µm had significantly longer disease duration and significantly more advanced disability than those with greater pRNFL thickness (Table 3).

Multivariate logistic regression analysis identified EDSS score and disease duration as clinical predictors of reaching pRNFL thicknesses of ≤87 µm and ≤88 µm. The risk of reaching pRNFL thickness ≤87 µm increases by 46% for each increment point in EDSS score (adjusted odds ratio [OR] 1.46, 95% CI 1.18–1.80; *p* < 0.001). The risk of reaching pRNFL thickness ≤87 µm increases by 9% for each subsequent year of disease duration (adjusted OR 1.09, 95% CI 1.04–1.14; *p* < 0.001). For each increment in EDSS by a point, the risk of reaching pRNFL thickness ≤88 µm increases by 42% (adjusted OR 1.42, 95% CI 1.16–1.74; *p* < 0.001). For each subsequent year of the disease duration, the risk of reaching pRNFL thickness ≤88 µm increases by 7% (adjusted OR 1.07, 95% CI 1.02–1.12; *p* = 0.003). In multivariate regression analysis, no statistically significant predictors of GCIPL thickness <70 µm were found.

### 3.3. Diagnostic Accuracy of OCT Cut-Off Values

**Figure 1:**
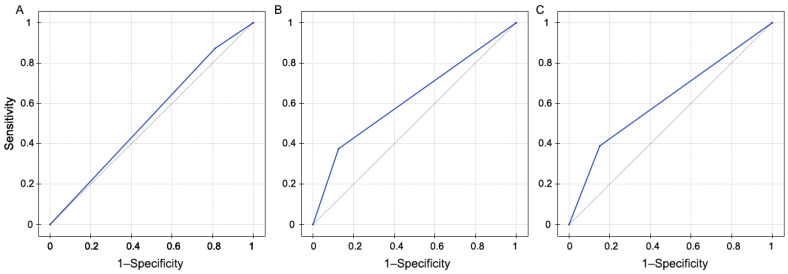
ROC curves for GCIPL thickness <70 µm (**A**), pRNFL thickness ≤87 µm (**B**) and ≤88 µm (**C**). ROC, receiver operating characteristic; GCIPL, ganglion cell–inner plexiform layer; pRNFL, peripapillary retinal nerve fiber layer.

**Figure 2:**
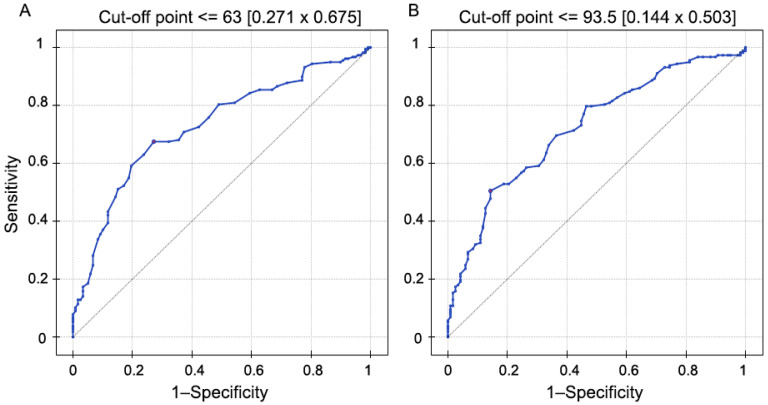
Optimal cut-off values for GCIPL (**A**) and pRNFL (**B**) thicknesses in the investigated cohort estimated by ROC analysis. ROC, receiver operating characteristic; GCIPL, ganglion cell–inner plexiform layer; pRNFL, peripapillary retinal nerve fiber layer.

In distinguishing pwMS with EDSS ≥ 3 from those with less severe disability, GCIPL thickness <70 µm yielded a sensitivity, specificity, accuracy, positive predictive value (PPV) and negative predictive value (NPV) of 87.3%, 18.5%, 57.8%, 58.8% and 68.3%, respectively. For discrimination pwMS with EDSS ≥ 3 from those with less severe disability, pRNFL thickness ≤87 µm provided a sensitivity, specificity, accuracy, PPV and NPV of 37.6%, 87.3%, 58.9%, 79.7% and 51.2%, respectively. While differentiating pwMS with EDSS ≥ 3 from those with less severe disability, pRNFL thickness ≤88 µm yielded a sensitivity, specificity, accuracy, PPV and NPV of 38.9%, 84.8%, 58.6%, 77.2% and 51%, respectively (Figure 1). There were significant differences between ROC curve for GCIPL < 70 µm and ROC curve for both pRNFL ≤ 87 µm as well as ≤88 µm (*p* = 0.005 and *p* = 0.004, respectively). No significant differences were found between the ROC curves of the two adopted pRNFL cut-offs (*p* = 0.458). The optimal cut-off value differentiating disability outcomes in our group estimated by ROC analysis was 63 µm for GCIPL thickness and 93.5 µm for pRNFL thickness with AUCs of 72.7% and 72.2%, respectively (Figure 2).

## 4. Discussion

We conducted a cross-sectional study to evaluate the usefulness of pRNFL and GCIPL thickness thresholds for single-time-point SS-OCT measurements in differentiating clinical outcomes in the real-world cohort of treatment-naïve pwMS without ON history. We found that for pRNFL thickness thresholds (87 µm and 88 µm, respectively), significant differences were observed in relation to the disease type. Importantly, no CIS patient had pRNFL thickness ≤87 µm. Furthermore, patients with pRNFL ≤87 µm as well as ≤88 µm had significantly longer disease duration and more advanced disability compared to those with greater pRNFL thickness. Importantly, such differences have not been established for GCIPL thickness thresholds (70 µm and 77 µm, respectively). Firstly, we had to exclude the GCIPL thickness cut-off point of 77 µm for statistical reasons, as lower values were found in as many as 270 patients (98%). Secondly, for the only adopted GCIPL thickness cut-off value of 70 µm, no significant differences were found in terms of the disease type, its duration, and disability level. In distinguishing pwMS with EDSS ≥3 from those with less severe disability, GCIPL thickness <70 µm had the highest sensitivity, while pRNFL thickness ≤87 µm had the greatest specificity. Disease duration and EDSS score were identified as clinical predictors of reaching pRNFL thickness ≤87 µm and ≤88 µm but not GICPL thickness <70 µm. The optimal cut-off value differentiating disability outcomes in our group estimated by ROC analysis was 63 µm for GCIPL thickness and 93.5 µm for pRNFL thickness. Notably, by excluding the influence of the ON history and the use of DMTs, we were able to more reliably assess retinal layer thinning related to MS-specific neurodegenerative processes using SS-OCT.

Generally, GCIPL thickness is considered a more clinically useful MS biomarker than pRNFL thickness due to superior structure–function relationships [13,23]. In line with these data, our study showed the highest sensitivity of the GCIPL cut-off of 70 µm in non-ON eyes for differentiating patients with no or minimal disability from those with at least moderate disability (EDSS ≥ 3) [32]. However, the adopted pRNFL thickness cut-offs (87 µm and 88 µm, respectively), and not the GCIPL thickness cut-off of 70 µm, measured at the single-time-point, differentiated our study population in terms of disease variant, its duration and disability status. Importantly, when referring our findings to previous studies, potential measurement differences for the retinal layer thickness thresholds should be indicated, which largely depend on the inclusion of pwMS with different clinical profiles in the study cohorts and the performance of tests using various generations of OCT devices. At this point, we should emphasize that the adopted GCIPL and pRNFL thickness thresholds were previously determined using SD-OCT and not SS-OCT devices.

Cut-off values of pRNFL thickness (≤87 µm and ≤88 µm) as well as GIPL thickness (<70 µm and <77 µm) have been cross-sectionally established as biomarkers of MS-related disability worsening within subsequent years [12,13,14,18]. Importantly, some studies analyzed only pRNFL thickness cut-offs, others only GCIPL thickness cut-offs, while some researchers considered the cut-off values of both retinal regions’ thicknesses using different SD-OCT devices. However, the cohorts assessed in these studies had a lower variety of MS types and less severe disability compared to our group, and retinal layer thickness was not assessed by using SS-OCT. Furthermore, in the studies mentioned above, most of the patients used DMTs, and the follow-up periods and the definition of disability progression varied between them [12,13,14,18]. Martinez-Lapiscina et al. +identified pRNFL thickness ≤87 μm or ≤88 μm (depending on the OCT device used) in non-ON eyes of pwMS as predictors of 5-year disability progression. Interestingly, no such associations were found for macular volume. The authors assessed 879 pwMS (74 CIS, 664 RRMS, 83 SPMS, 58 PPMS) with a median EDSS score of 2, 557 of whom (63%) used DMTs. In the context of a more significant association of pRNFL thickness than macular volume in non-ON eyes with disability outcomes, these results seem consistent with our findings [12]. The importance of cross-sectional monitoring of pRNFL thinness was also emphasized by Bsteh et al. In this study, pRNFL thickness ≤88 µm in non-ON eyes was independently associated with a three-fold increased risk of disability progression and a 2.7-fold increased risk of cognitive decline within the subsequent 3 years in 151 RRMS patients with a median baseline EDSS score of 1.5. Notably, the exposure to DMTs experienced by 90 patients (59.6%) in this group had a significant protective effect on pRNFL thinning [18]. In our study, relationships between pRNFL thickness cut-offs and disability outcomes were also confirmed by identifying clinical predictors of reaching values ≤87 µm and ≤88 µm, the strongest of which was the EDSS score. Similar to our findings, Skirková et al. found a significant association of thinner pRNFL in non-ON eyes of pwMS (median EDSS of 3.5) with a more advanced disability but did not reveal such a relationship for ganglion cell complex thickness [33].

Although growing evidence supports the superiority of GCIPL thickness over pRNFL thickness as a biomarker of disability progression in pwMS, some of them concern only cohorts of RRMS patients using DMTs [34]. Furthermore, it has been shown that GCIPL thickness corresponded better with DMTs failure than pRNFL thickness in RRMS patients [35]. We should emphasize that our study does not discredit GICPL thickness as a valid parameter that significantly differentiates MS variants, disease duration and patients’ disability. It has been demonstrated that GCIPL thinning occurs in the early stages of MS, even when pRNFL thickness remains within the normal range [36]. Therefore, GCIPL atrophy is an early and sensitive marker of MS-related neurodegeneration and disability progression. As revealed in our study, the higher sensitivity of GICPL thickness <70 µm compared to both adopted pRNFL thickness cut-offs in distinguishing pwMS with moderate disability from those with no or minimal disability is consistent with the data above. However, we are aware that the adopted pRNFL and GCIPL thickness thresholds were previously determined using various SD-OCT devices, and may not fully coincide with the measurements obtained using SS-OCT.

In our group, however, mean GCIPL thickness was below the adopted cut-off (an average of 7 µm), while mean pRNFL thickness in both eyes was above the adopted cut-off points (an average of 9–10 µm). With such a low mean GCIPL thickness, the cut-off <70 µm could not reliably differentiate pwMS in terms of clinical outcomes, as almost 85% of patients had a lower average value of this parameter. We realize that the discrepancies in pRNFL and GCIPL thickness in relation to the adopted cut-offs had to translate into their clinical significance. We postulate that cohorts containing patients with different MS variants may pose a challenge in establishing the optimal OCT cut-off values of the neurodegenerative sensitive macular region for differentiating patients in terms of selected MS outcomes, as previously demonstrated in the study by Martinez-Lapiscina et al. [12]. Thus, we determined optimal SS-OCT cut-off values for differentiating disability outcomes in our cohort characterized by a good diagnostic accuracy of 63 µm for GCIPL thickness and 93.5 µm for pRNFL thickness (AUC of 72.7% and 72.2%, respectively). A diagnostic test with AUC between 0.90 and 1.00 has excellent discrimination ability, while AUC from 0.80 to 0.90, 0.70 to 0.80, 0.60 to 0.70, and 0.50 to 0.60 indicates very good, good, sufficient and bad discrimination ability, respectively [37,38]. Lambe et al. determined that mean baseline GCIPL thickness <70 μm in non-ON eyes measured by SD-OCT was associated with an increased risk of long-term disability worsening in 132 pwMS. The authors used the cut-off value for GCIPL thickness of 70 μm as it represented the mean baseline GCIPL thickness of all eligible patients’ eyes. Notably, the optimal GCIPL thickness cut-off differentiating disability outcomes estimated for our group was also equal to its mean value, which may support the validity of the concept used by Lambe and colleagues. Interestingly, in the study by Lambe et al., pwMS with an EDSS of 3 had an average GCIPL thickness of 63.78 µm, which is also consistent with the measurements obtained in our study despite the use of different OCT devices [13].

The presented study revealed that none of the CIS patients had pRNFL ≤87 µm, which may indicate the usefulness of this cut-off in distinguishing non-ON CIS eyes from non-ON MS eyes. Interestingly, no such differences were found for the adopted GCIPL thickness cut-off value of 70 µm. The majority of our patients, regardless of the disease type, had an average GCIPL thickness <70 µm. Our results confirm previous reports on neuro-retinal changes reflecting neurodegeneration within the CNS from the earliest disease stages in non-ON eyes [39]. Typically, GCIPL atrophy reflects neuroaxonal damage faster than pRNFL thinning, as reflected in our group [7,40]. Therefore, pRNFL thickness ≤87 µm may be a potential cut-off point differentiating CIS non-ON eyes from MS non-ON eyes. Although these findings indicate the potential utility of retinal layer thickness thresholds in new diagnostic areas, they need to be interpreted with caution due to the limited number of CIS patients in our group (n = 23) and should be confirmed in cohorts containing more CIS patients using SS-OCT devices.

The strength of our study is the relatively large sample size of treatment-naïve pwMS without previous subclinical/clinical ON. These data are particularly important when analyzing the impact of the single-time-point OCT measurements on MS-related neurodegeneration independent of DMTs and ON history. Furthermore, we used SS-OCT imaging, the newest generation of OCT machines that allow greater retinal tissue penetration compared to SD-OCT [41]. Although SS-OCT is a promising diagnostic approach in pwMS, pRNFL and GCIPL thickness cut-offs have not yet been evaluated using such devices [42]. Thus, the presented study is the first to analyze the thresholds for single-time-point SS-OCT measurements in evaluating MS clinical outcomes. The use of thresholds for single-time-point SS-OCT measurements may be a valuable complement to clinical decision support systems in a holistic approach to pwMS [43]. At the same time, we are aware of our study limitations. The cut-off points for GCIPL and pRNFL thicknesses associated with disability status adopted in our study were previously established using SD-OCT devices [12,13,14]. Moreover, we performed only single-time-point assessments of pRNFL thickness, GCIPL thickness, and EDSS score without longitudinal evaluation of retinal thinning or disability progression. Another limitation was the lack of assessment of the relationships between neurodegeneration markers obtained from OCT measures with visual field, visual acuity, and MRI findings. Furthermore, our assumptions concerning non-ON eyes have not been confirmed by the absence of demyelinating lesions on MRI scans of the optic nerves. Finally, we did not evaluate the inner plexiform layer thickness assessment, which was reported as a promising biomarker of MS inflammatory activity and relapse [14].

## 5. Conclusions

In the present study, we demonstrated the usefulness of pRNFL and GCIPL thickness thresholds for single-time-point SS-OCT measurements in non-ON eyes in differentiating clinical outcomes among treatment-naïve pwMS. GCIPL thinning in non-ON eyes has emerged as an early and sensitive biomarker of MS-related neurodegeneration reflected in treatment-naïve patients’ disabilities. The cut-off values of pRNFL thickness in non-ON eyes were helpful in differentiating clinical outcomes in pwMS and in discriminating CIS non-ON eyes from non-ON MS eyes. However, although we determined the optimal pRNFL and GCIPL thickness cut-off values for differentiating disability status using SS-OCT, it should be noted that, for the baseline analysis of clinical outcomes, we adopted the thresholds previously established using SD-OCT devices and did not correlate our findings with MRI data. Further studies are needed to show another possible usefulness of pRNFL and GCIPL thickness thresholds for SS-OCT measures with the determination of specific values in the new diagnostic indications, considering cohorts with a diverse clinical profile of pwMS and using both single-time-point and follow-up assessment.

## Figures and Tables

**Table 1:** Baseline characteristics.

MS Patients	Statistical Parameters				
	N (%)	M	SD	95% CI	Min.–Max.
Gender					
Female	189 (69%)				
Male	86 (31%)				
Disease type					
CIS	23 (9%)				
BNMS	8 (3%)				
PPMS	31 (11%)				
RRMS	185 (67%)				
SPMS	28 (10%)				
Age (years)		41.3	12.4	39.9–42.8	18–72
Disease Duration (years)		6.4	7.1	5.6–7.3	0–46
EDSS score		3.0	1.6	2.8–3.2	0–7.0
pRNFL thickness (total eyes)		97.1	11.7	95.7–98.5	61–128
pRNFL thickness (right eyes)		97.4	12.3	96.0–98.9	62–128
pRNFL thickness (left eyes)		96.9	12.4	95.4–98.4	61–127
GCIPL thickness (total eyes)		63.0	7.0	62.2–63.9	43–83
GCIPL thickness (right eyes)		63.0	7.1	62.1–63.8	45–82
GCIPL thickness (left eyes)		63.1	7.4	62.2–64	43–83

The number (N), percentage (%), mean (M), standard deviation (SD), 95% confidence interval (CI) and minimum-to-maximum values are shown. CIS, clinically isolated syndrome; BNMS, benign multiple sclerosis; RRMS, relapsing–remitting multiple sclerosis; SPMS, secondary progressive multiple sclerosis; PPMS, primary progressive multiple sclerosis; EDSS, Expanded Disability Status Scale; pRNFL, peripapillary retinal nerve fiber layer; GCIPL, ganglion cell–inner plexiform layer.

**Table 2:** Characteristics of the study cohort by cut-off values of pRNFL and GCIPL thickness (discrete variables) (n = 275).

MS Patients	Gender (N,%)		*p*-Value	Disease Type (N,%)					*p*-Value
	Female	Male		CIS	BNMS	RRMS	SPMS	PPMS	
pRNFL thickness ≤87 µm (n = 74) >87 µm (n = 201)	46 (62%) 143 (71%)	28 (38%) 58 (29%)	0.154	0 23 (11%)	1 (1%) 7 (4%)	43 (58%) 142 (71%)	16 (22%) 12 (6%)	14 (19%) 17 (8%)	<0.001
pRNFL thickness ≤88 µm (n = 79) >88 µm (n = 196)	51 (65%) 138 (70%)	28 (35%) 58 (30%)	0.344	1 (1%) 22 (11%)	1 (1%) 7(4%)	47 (60%) 138 (70%)	16 (20%) 12 (6%)	14 (18%) 17 (9%)	<0.001
GCIPL thickness <70 µm (n = 233) ≥70 µm (n = 42)	165 (71%) 24 (57%)	68 (29%) 18 (43%)	0.114	19 (8%) 4 (10%)	7 (3%) 1 (2%)	158 (68%) 27 (64%)	21 (9%) 7 (17%)	28 (12%) 3 (7%)	0.544

MS, multiple sclerosis; CIS, clinically isolated syndrome; BNMS, benign multiple sclerosis; RRMS, relapsing–remitting multiple sclerosis; SPMS, secondary progressive multiple sclerosis; PPMS, primary progressive multiple sclerosis; pRNFL, peripapillary retinal nerve fiber layer; GCIPL, ganglion cell–inner plexiform layer.

**Table 3:** Characteristics of the study cohort by GCIPL and pRNFL cut-off values (numerical variables) (n = 275).

MS Patients	Cut-Off Point	Statistical Metrics				*p*-Value
		M	SD	95% CI	Min.–Max.	
Age (years)	GCIPL <70 µm ≥70 µm	41.1 42.7	12.1 14.5	39.5–42.7 38.2–47.2	18–72 18–71	0.440
	pRNFL ≤87 µm >87 µm	41.3 41.4	12.1 12.6	38.5–44.1 39.6–43.1	19–69 18–72	0.821
	pRNFL ≤88 µm >88 µm	41.1 41.4	11.8 12.7	38.5–43.8 39.6–43.2	19–69 18–72	0.7
Disease Duration (years)	GCIPL <70 µm ≥70 µm	6.4 6.2	7.2 6.8	5.5–7.4 4.1–8.4	1–33 0–46	0.818
	pRNFL ≤87 µm >87 µm	9.5 5.3	7.8 6.5	7.7–11.3 4.4–6.2	1–33 0–46	<0.001
	pRNFL ≤87 µm >88 µm	9.1 5.3	7.7 6.6	7.3–10.8 4.4–6.3	1–33 0–46	<0.001
EDSS	GCIPL <70 µm ≥70 µm	3.0 3.0	1.6 1.7	2.8–3.2 2.5–3.5	0–7.0 0–6.5	0.872
	pRNFL ≤87 µm >87 µm	3.8 2.8	1.4 1.6	3.5–4.1 2.5–3.0	1.0–7.0 0–6.5	<0.001
	pRFNL ≤88 µm >88 µm	3.7 2.8	1.4 1.6	3.4–4.0 2.5–3.0	0–7.0 0–6.5	<0.001

The number (N), percentage (%), mean (M), standard deviation (SD), 95% confidence interval (CI) and minimum-to-maximum values are shown. MS, multiple sclerosis; EDSS, Expanded Disability Status Scale; pRNFL, peripapillary retinal nerve fiber layer; GCIPL, ganglion cell–inner plexiform layer.

## Data Availability

The datasets generated during and/or analyzed during the current study are available from the corresponding author on reasonable request.

## Abbreviations

Acronym	Meaning
ANOVA	analysis of variance
APOSTEL	Advised Protocol for Optical Coherence Tomography Study Terminology and Elements
AUC	area under the curve
BNMS	benign multiple sclerosis
CI	confidence interval
CIS	clinically isolated syndrome
CNS	central nervous system
DMTs	disease-modifying treatments
EDSS	Expanded Disability Status Scale
GCIPL	ganglion cell–inner plexiform layer
LSD	least significant differences
MS	multiple sclerosis
non-ON	non-optic neuritis
NPV	negative predictive value
OCT	optical coherence tomography
ON	optic neuritis
OR	odds ratio
PPMS	primary progressive multiple sclerosis
PPV	positive predictive value
pRNFL	peripapillary retinal nerve fiber layer
pwMS	people with MS
ROC	receiver operating characteristic
RRMS	relapsing–remitting multiple sclerosis
SD	standard deviation
SD-OCT	spectral-domain optical coherence tomography
SPMS	secondary progressive multiple sclerosis
SS-OCT	swept-source optical coherence tomography
